# Supplementary material for: Predictive factors associated with bleeding in atrial fibrillation patients treated with anti-coagulant drugs using a large claims database
Source: PLoS One. 2020 Aug 31;15(8):e0238233. doi: 10.1371/journal.pone.0238233 (PMC7458330; doi:10.1371/journal.pone.0238233)
Supplement: S1 Table — (DOCX) [file pone.0238233.s001.docx]

|  |  |  |  | Without cerebrovascular disease (n=2499) | | | |  | With cerebrovascular disease (n=297) | | | |
| --- | --- | --- | --- | --- | --- | --- | --- | --- | --- | --- | --- | --- |
|  |  |  |  | With bleeding | Without bleeding | Adjusted Odds ratio (95% CI) | *p* value |  | With bleeding | Without bleeding | Adjusted Odds ratio (95% CI) | *p* value |
|  | Sex, number | | Male | 222 | 1901 | Reference |  |  | 59 | 192 | Reference |  |
|  |  |  | Female | 72 | 304 | 1.76 (1.29-2.41) | 0.0004 |  | 18 | 28 | 1.93 (0.90-4.11) | 0.0893 |
|  | Age, average [SD], (+1 year) | |  | 58.1±9.9 | 55.3±9.6 | 1.03 (1.02-1.05) | <0.0001 |  | 60.5±9.4 | 58.9±93 | 1.03 (0.99-1.07) | 0.1099 |
|  | Number of co-administered drugs, median with range, (+1 drug) | |  | 4 (1-18) | 4 (1-18) | 1.07 (1.03-1.12) | 0.0026 |  | 7 (1-15) | 5 (1-15) | 1.13 (1.02-1.25) | 0.0153 |
|  | Anticoagulant agent, number | | DOAC | 168 | 1441 | Reference |  |  | 33 | 138 | Reference |  |
|  |  |  | Warfarin | 126 | 764 | 1.29 (0.99-1.69) | 0.0575 |  | 44 | 82 | 2.60 (1.39-4.89) | 0.0029 |
|  | Co-administration of anti-platelet drug, number | | Without | 273 | 2107 | Reference |  |  | 60 | 184 | Reference |  |
|  |  |  | With | 21 | 98 | 1.12 (0.64-1.96) | 0.6911 |  | 17 | 36 | 1.66 (0.78-3.56) | 0.1896 |
|  | Drug-drug interaction, number | | Without | 274 | 2129 | Reference |  |  | 70 | 210 | Reference |  |
|  |  |  | With | 20 | 76 | 1.72 (0.99-3.00) | 0.0562 |  | 7 | 10 | 1.81 (0.55-5.92) | 0.3278 |
|  | Past history of bleeding, number | | Without | 252 | 2184 | Reference |  |  | 48 | 201 | Reference |  |
|  |  |  | With | 42 | 21 | 13.92 (8.01-24.20) | <0.0001 |  | 29 | 19 | 9.24 (4.31-19.78) | <0.0001 |
| Number of co-administered drugs × Age | | |  |  |  |  | 0.0208 |  |  |  |  | 0.0121 |

**S1 Table. Adjusted odds ratio for co-factors associated with bleeding after administration of anti-coagulants using multiple logistic regression analysis stratified based on presence or absence of cerebrovascular disease.**
