# Supplementary material for: Predictive factors associated with bleeding in atrial fibrillation patients treated with anti-coagulant drugs using a large claims database
Source: PLoS One. 2020 Aug 31;15(8):e0238233. doi: 10.1371/journal.pone.0238233 (PMC7458330; doi:10.1371/journal.pone.0238233)
Supplement: Attachment — Submitted filename: Revise_letter_20200403KM.docx [file pone.0238233.s002.docx]

**To Reviewer 1**

1. The authors concluded that the interaction term for “number of co-administered drugs * age” was identified as a predictive factor for bleeding, the evaluation of which was conducted with logistic regression. The logistic regression model adopted for patients without cerebrovascular disease includes several variables such as age and number of co-administered drugs, along with the interaction term. The p-value of the interaction term (p=0.0208) does represent that the interaction is significant, which indicates that the effect of number of co-administration drugs on bleeding is different at different values of age, but does not represent that the interaction term is a significant predictive factor. The same criticism is applicable for the model for patients with cerebrovascular disease. (The above are based on my recognition. Further review by statistician might be preferable.). Although the authors conducted detailed analysis to show that the bleeding risk increased with the increase of concomitant drug administration in younger patients, but did not in elderly patients, the data were not shown. Because these results are declared in the ABSTRACT, the data are so important that they should be presented. The data are also important to validate the interaction term and the model itself eventually.

**We appreciate your pertinent observations and suggestions. A co-author, Dr. Koinuma is a statistician, and he analysed our data. According to the suggestion, we added the analysis in table 3 and added the description (line 33-39, 138-141, 216-229, 244-250).**

**In general, if interactions are found in categorical variables (eg. Sex), stratified analysis is conducted. In our study, interactions occurred between the variables used, namely, age (continuous variable) and the number of co-administered drugs (continuous variable). Therefore, we presented the results descriptively, as continuous variables usually need to be analyzed as continuous variables.**

**However, Kokkinos P et al. (Lancet 2013; 381: 394-99) reported that on changing continuous variables to categorical variables (MET: metabolic equivalents), they found interactions. In accordance with their analysis, we also changed the continuous variables to categorical variables (age cut off value: 60) (Table 3). The age of 60 years was selected based on the time for drastic changes in the working environment such as retirement, position retirement, or employment status (Table 3). In order to avoid over fitting, we added 4 factors (sex, the number of co-administration drugs, anticoagulant agent, and past history of bleeding) to our model in table 3.**

2. Adequate explanation for the validity of the logistic regression models, especially for the reason for the selection of the variables, is needed, as the authors selected these variables amongst plenty of candidate variables in large claims database.

**As suggested, we evaluated the model validity using lack of fitness. In the basic model (Table 2), the fit was adequate. On stratified analysis (Table 3), the value did not fit. We have mentioned this in the manuscript at the method and limitation area (line 143-144, 287-291).**

**As suggested, the reasons for selecting the included variables have also been mentioned in the manuscript (line 127-132).**

3. The entry criteria, referred in Results section, is not specified elsewhere.

**We changed the term “entry criteria” to “case identification flow” to improve clarity (line 159).**

3. Improvements to the English language within the manuscript is desired.

E.g.;

L.27

... after stratification without and with cerebrovascular diseases...

L.145

With bleeding patients, prescription pattern for anticoagulants were high for warfarin, but lower for DOACs.

L.182 Table 2.

Adjusted odds ratio for co-factors associates bleeding after administration of anti-coagulants using multiple logistic regression analysis stratified with and without cerebrovascular disease.

cerebovascular -> cerebrovascular

And so on.

**As suggested, we have revised throughout the manuscript, and had it re-edited by a professional English language editing service (Editage). Revised point was wrote in red in the manuscript.**

**To Reviewer 2**

#Major issues

The authors described as “The interaction term showed that, in young patients, the risk for bleeding was greater as the number of co-administered drugs increased. By contrast, in elderly patients, the risk for bleeding did not increase with increasing number of co-administered drugs (Table 2)” (p.12, L.178-181）. This result is an important finding in this manuscript, but not enough data has been provided to support it. The supporting data should be presented in a table or figure.

**We appreciate your pertinent observations and suggestion, and have revised the manuscript accordingly; the stratification in the elderly and young has been included as the second stratified analysis in Table 3 and added the description (line: 33-39, 138-141, 216-229, 244-250).**

**In general, if interactions are found in categorical variables (eg. Sex), stratified analysis is conducted. In our study, interactions occurred between the variables used, namely, age (continuous variable) and the number of co-administered drugs (continuous variable). Therefore, we presented the results descriptively, as continuous variables usually need to be analyzed as continuous variables.**

**However, Kokkinos P et al. (Lancet 2013; 381: 394-99) reported that on changing continuous variables to categorical variables (MET: metabolic equivalents), they found interactions. In accordance with their analysis, we also changed the continuous variables to categorical variables (age cut off value: 60) (Table 3). The age of 60 years was selected based on the timing for drastic changes in the working environment such as retirement, position retirement, or employment status (Table 3). In order to avoid over fitting, we added 4 factors (sex, the number of co-administration drugs, anticoagulant agent, and past history of bleeding) to our model in table 3.**

#Minor issues

In table 2, odds ratio of “number of co-administration drugs*Age” should be described.

**The odds ratio for the interaction terms could not be calculated as the changes in the continuous variable * continuous variable values were complex.**

**As suggested, we added the details of the second stratified analysis for age in the young and elderly to table 3. The odds ratio for the number of co-administered drugs in the elderly (age ≥ 60, ≤74) was 1.05 [0.99 – 1.12], N.S. in the group without cerebrovascular disease and 1.10 [0.96 – 1.27], N.S. in the group with cerebrovascular disease. In contrast, the values in the young were 1.09 [1.03 – 1.16], p=0.0054 in the group without cerebrovascular disease and 1.20 [1.05 – 1.36], p=0.0059 in the group with cerebrovascular disease (Table 3). We add the description (line 33-39, 138-141, 216-229, 244-250).**

#Other comments

Is the description of “Direct vitamin K inhibitors (DOAC)”（ｐ.4, L.50）correct ?

**As suggested, we have revised this to “Direct Oral Anti-Coagulant” (line 50).**

Is the symbol in “p>0.0001” (p.9, L.146) correct ?

**We apologize for the error and have revised this from “>” to “<” (line 161).**

Is the number of “2800” (p.11, L.127) correct ?

**We apologize for the error and have revised this from “2800” to “2796” (line 191).**

References No.8 and No.12 are the same, and their descriptions are duplicated.

**We apologize for the error and have removed ref. 12 and add ref. 8; the reference numbers have also been changed throughout the manuscript.**

**To Reviewer 3**

Reviewer #3: I read the paper of “Predictive factors associated with bleeding in atrial fibrillation patients treated with anti-coagulant drugs using a large claims database” interestingly. I was particularly interested in discussing the relationship between age and the number of co-administered drugs as predictors of bleeding. I recognized that it was a very useful paper in terms of polypharmacy and medical safety.

Please consider the following points.

Please correct "Shu-toh Kana" to "Kana Shu-toh" in the author part.

**We apologize for the error, and have changed "Shu-toh Kana" to "Kana Shu-toh" (line 5).**

How old should elderly patients be considered in this paper? I think it is better to show a specific age in the discussion.

**As suggested, we defined elderly individuals as those aged ≥ 60. We have added the details of the analysis for stratification in the young and elderly to table 3, and have added the results of the second stratified analysis for age (in the young and elderly) to table 3. The odds ratio for the number of co-administered drugs in the elderly (age ≥ 60, ≤74) was 1.05 [0.99 – 1.12], N.S. in the group without cerebrovascular disease and 1.10 [0.96 – 1.27], N.S. in the group with cerebrovascular disease. In contrast, the values in the young were 1.09 [1.03 – 1.16], p=0.0054 in the group without cerebrovascular disease and 1.20 [1.05– 1.36], p=0.0059 in the group with cerebrovascular disease (table 3). And we added the description (line 33-39, 138-141, 216-229, 244-250).**
