# Supplementary material for: Predictive factors associated with bleeding in atrial fibrillation patients treated with anti-coagulant drugs using a large claims database
Source: PLoS One. 2020 Aug 31;15(8):e0238233. doi: 10.1371/journal.pone.0238233 (PMC7458330; doi:10.1371/journal.pone.0238233)
Supplement: Attachment — Submitted filename: R2 Responses to the Reviewer 20200716KM.docx [file pone.0238233.s003.docx]

**Responses to the Reviewer’s comments (PONE-D-20-00210R1)**

We would like to express our gratitude to the reviewers for their insightful comments on our paper. We feel that these comments have helped us to improve the manuscript substantially. Our point-by-point responses to the reviewer’s comments can be found below:

**Responses to Reviewer:**

**Comment #1-1**

The authors added Table 3 and related description according to stratified analysis by the age of 60 years. The revision is to be appropriate, as these results are quite important to support their opinion.

However, this revision generated another concern; Is Table 2 necessary? The authors conducted stratified analysis because interactions are found. Therefore, the predictive factors should be discussed based on the stratified analysis, as authors have done so.

**Response:**

We appreciate your comment on this point. We removed table 3 and changed it to suppl

1. According to removing table 3, we revised abstract (p2, l34-50), Method (p7, l 147-

p8, l 149), Result (p12, l 212-232) and Discussion (p18, l265-268, p19, l286-p20, l308, p19, l319-321).

**Comment #1-2**

No explanation is provided about the predictive factors that I have mentioned. i.e. Is the sentence in ABSTRACT, “Multiple logistic regression analysis revealed the following cerebrovascular disease associated predictors of bleeding in both groups: ...3) interaction term for “number of co-administrered drugs × age”” correct? I don’t think the expression correct. Is regarding an interaction term as a predictor accurate?

**Response:**

We appreciate your comment on this point. According to the suggestion for comment

#1-1, we removed table 3 and mentioned for only the stratified analysis (young and

elderly) data in revised manuscript. We showed the predictive factor as "history of

bleeding" in young and elderly, and "polypharmacy" and "start from warfarin" in young

in revised manuscript (p2, l43-44, p12, l227-232).

**Comment #2**

Explanations for the validity of their regression models and the reasons for selecting variables are thought to be properly added.

**Response:**

Thank you for your comment.

**Comment #3**

The term “case identification flow” is to be appropriate.

**Response:**

Thank you for your comment.
