# Supplementary material for: Predictive factors associated with bleeding in atrial fibrillation patients treated with anti-coagulant drugs using a large claims database
Source: PLoS One. 2020 Aug 31;15(8):e0238233. doi: 10.1371/journal.pone.0238233 (PMC7458330; doi:10.1371/journal.pone.0238233)
Supplement: Attachment — Submitted filename: R3 Responses to the Reviewer 20200810KM.docx [file pone.0238233.s004.docx]

**Responses to the Reviewer’s comments (PONE-D-20-00210R1)**

We would like to express our gratitude to the reviewers for their insightful comments on our paper. We feel that these comments have helped us to improve the manuscript substantially. Our point-by-point responses to the reviewer’s comments can be found below:

**Responses to Reviewer:**

Thank you for editing English expression. We revised according to the suggestion.

**Comment #1-1**

Abstract

p.2 l.34

Bleeding risk assessment using multiple logistic regression analysis, the odds ratio for the number of co-administered drugs in the elderly (age for ≥60, ≤74) was not significant in those without and with cerebrovascular diseases ...

->

In bleeding risk assessment using multiple logistic regression analysis, …

Or

Bleeding risk assessment using multiple logistic regression analysis revealed that the odds ratio…

**Response:**

According to the suggestion, we revised to “….multiple logistic regression analysis revealed that the odds ratio….”

**Comment #1-2**

Results

p.12 l.227

Data shown in first order analysis, we stratified…

->

Based on data shown in first order analysis, we stratified…

**Response:**

According to the suggestion, we revised to “Based on data ….”

**Comment #1-3**

Discussion

p.19 l.267

We identified the predictive factors for bleeding after starting anticoagulants in AF patients among young and elderly and presence and absence of cerebrovascular disease, only the history of bleeding was a risk factor in the elderly, but in the young, polypharmacy, starting with warfarin, and a history of bleeding were the observed potential predictive factors for bleeding.

->

We identified the predictive factors for bleeding after starting anticoagulants in AF patients among young and elderly both in the presence and absence of cerebrovascular disease. In the elderly, only the history of bleeding was a risk factor but in the young, polypharmacy, starting with warfarin, and a history of bleeding were the observed potential predictive factors for bleeding.

**Response:**

According to the suggestion, we revised to “…among young and elderly both in the presence and absence of cerebrovascular disease. In the elderly, only the history of bleeding was a risk factor but in the young,…”
